# Networking of psychophysics, psychology, and neurophysiology

**DOI:** 10.3389/fphys.2012.00423

**Published:** 2012-11-05

**Authors:** Bruce J. West, Paolo Grigolini

**Affiliations:** ^1^Information Science Directorate, US Army Research Office, Research Triangle ParkNC, USA; ^2^Center for Nonlinear Science, University of North Texas, DentonTX, USA

The Army Research Laboratory program on the *Network Science of Human Decision Making* brought together researchers from a variety of disciplines to work on a complex research problem that defies confinement within any single discipline. Consequently, new and rewarding solutions have been obtained for problems of importance to society and the Army in the human dimension of complex networks. This program is reviewed by West (2011) in *Overview 2010 of ARL program on network science for human decision making* wherein he investigates the basic research foundation of a science of networks supporting the linkage between the cognitive and social domains as they relate to human decision making. In the same spirit as the present workshop the research strategy extends recent methods of non-equilibrium statistical physics to non-stationary, renewal stochastic processes characteristic of the interactions among nodes in complex networks. The theoretical analyses of complex networks, although mathematically rigorous, often elude analytic solutions and require simulation and computation to analyze the underlying dynamic process.

Dynamic networking and dynamic networks suggest new ways to transfer information utilizing the long-distance communication through local cooperative interaction. The papers contributed to the workshop clarified how the global intelligence of a complex network emerges from the local cooperation of units, whether these units are neurons or people and emphasizes the role played by critical phase transitions in the observed persistence of this cooperation.

To many scientists the gap between the nineteenth century views of consciousness proposed by the psychologist William James and that developed by the inventor of psychophysics Gustav Fechner has never seemed wider. However the chasm may not be as large as believed as (Hawkins, 2011) explains in *William James, Gustav Fechner, and Early Psychophysics*. The twenty-first century concept of collective/cooperative behavior within the brain has partially reconciled these diverging perspectives suggesting the notion of consciousness as a physical phenomenon as so eloquently explained by the late Gerhard Werner (Werner, 2011) in *Letting the Brian Speak for Itself*. He recognized that the self-referential mode of function and the propensity for self-organization to critical states requires a fundamentally new orientation, based on Complex System Dynamics as non-ergodic, non-stationary processes with inverse-power-law statistical distributions.

According to an increasing number of researchers intelligence emerges from criticality as a consequence of locality breakdown and the onset of long-range correlation, well-known properties of phase transition processes. Turalska et al. (2012) in *Cooperation-induced topological complexity: a promising road to fault tolerance and Hebbian learning* study a model of interacting units, as an idealization of real cooperative systems such as the brain or a flock of birds, for the purpose of discussing the emergence of long-range correlation from the coupling of any unit with its nearest neighbors. They focus on the critical condition that has been recently shown to maximize information transport and study the topological structure of the network of dynamically linked nodes.

Some investigators base the focus of their discussion on understanding the apparent contamination of social dynamics by erratic fluctuations. *Social interactions model and adaptability of human behavior* by Zhao and Bianconi (2011) concerns human social networks and their evolution on the fast timescale of face-to-face interactions and of interactions mediated by technology such as telephone calls and video conferences. The resulting networks have a strong dynamical component that changes significantly the properties of dynamical processes. They study a general model of pair wise human social interaction intended to model both face-to-face interactions and mobile-phone communication.

Fluctuations in social and brain dynamics, although apparently erratic, when analyzed with advanced methods of fractal statistical analysis reveal the emergence of complex behavior, intermediate between complete order and total randomness, a property usually referred to as temporal complexity. *Renormalization group for critical phenomena in complex networks* (Boettcher and Brunson, 2011) provides a detailed, pedagogical introduction to the application of renormalization group theory to the understanding of criticality in complex networks.

The physical singularity of life phenomena is analyzed in *The inert vs. the living state of matter: extended criticality, time geometry, anti-entropy – an overview* by Longo and Montévil (2012) by means of comparison with the driving concepts of theories of the inert. They outline conceptual analogies, transferals of methodologies and theoretical instruments between physics and biology, in addition to indicating significant differences and sometimes logical dualities. In order to make biological phenomenalities intelligible, they introduce theoretical extensions to certain physical theories such as criticality.

Others, with the help of modern technologies, such as functional magnetic resonance imaging (fMRI), establish a more direct analysis of brain dynamics, and focus on the brain's topological complexity. In *Criticality in large-scale brain fMRI dynamics unveiled by a novel point process analysis* (Tagliazucchi et al., 2012) introduces a theoretical framework in terms of an order and control parameter derived from fMRI data, where the dynamical regime can be interpreted as one corresponding to a system close to the critical point of a second order phase transition. The analysis demonstrates that the resting brain spends most of the time near the critical point of such transition and exhibits avalanches of activity ruled by the same dynamical and statistical properties described for neuronal events at smaller scales.

Neurophysiology research work has an increasing overlap with the emerging field of complex networks, and the behavior psychology experiments have until recently ignored the complex cooperative dynamics that are proved by increasing experimental evidence to characterize the brain function. Lovecchio et al. (2012) in *From self-organized to extended criticality* implemented the notion of extended criticality, which is realized through a wide set of critical points rather than emerging as a singularity from a unique value of the control parameter. Their approach explained the experimental observation that neuronal avalanches occur in time with surprisingly regularity, in apparent conflict with the temporal complexity of physical critical points.

Gallos et al. (2012) address the problem of the hierarchical organization in the brain through network analysis. In *The conundrum of functional brain networks: small-world efficiency or fractal modularity* their analysis identified functional brain modules of fractal structure that were inter-connected in a small-world topology. They provide details on the use of network science tools to elaborate on this network behavior and indicate the importance of using percolation theory to highlight the modular character of the functional brain network. These modules present a fractal, self-similar topology, identified through fractal network methods.
